# HLA-DR expression on monocytes is decreased in polytraumatized patients

**DOI:** 10.1186/s40001-015-0180-y

**Published:** 2015-10-16

**Authors:** Helen Vester, P. Dargatz, S. Huber-Wagner, P. Biberthaler, M. van Griensven

**Affiliations:** Department of Trauma Surgery, Klinikum rechts der Isar, Technical University Munich, Ismaninger Strasse 22, 81675 Munich, Germany

**Keywords:** Multiple trauma, SIRS, MODS, Sepsis, HLA-DR

## Abstract

**Background:**

Sepsis, systemic inflammatory response syndrome (SIRS) and multiple organ dysfunction syndrome (MODS) remain the most frequent causes of complications and death in severely injured patients. A main reason for the development of these syndromes is a post-traumatic dysregulation of the immune system. Several studies in intensive care unit (ICU) patients could detect a pivotal role of HLA-DR expression on monocytes. So far, its importance for development of SIRS, sepsis or MODS in the severely injured patient is not clear.

**Methods:**

Therefore, we have analysed HLA-DR expression on monocytes from severely injured patients (ISS > 16) during the post-traumatic course, which was on the day of trauma, as well as on days 3, 7 and 14 post trauma. Clinical data were analysed and the HLA-DR expression levels of patients who developed post-traumatic sepsis, SIRS or MODS were compared to those with a more favourable outcome. Young and healthy volunteers as well as patients undergoing prosthetic hip replacement after trauma were enrolled as control groups. HLA-DR molecules on monocytes were marked with PE-conjugated antibodies and the mean fluorescence intensity (MFI) was analysed via flow cytometry.

**Results:**

24 severely injured patients (mean age 34 ± 2.7 years) mainly after high energy motor vehicle accidents as well as 8 controls (total hip replacement) and 9 healthy volunteers (mean age 26.2 ± 1.2 years) were enrolled. A total of eight patients suffered from sepsis (33.3 %) (six males, two females) and 17 patients suffered from SIRS (70.9 %) (10 males, 7 females). MODS was present in five patients (20.8 %), three male and two female patients. In four of these five patients the MODS developed subsequent to sepsis. HLA-DR expression significantly decreased after trauma and slowly returned to normal after 14 days, irrespective of the complications developed.

**Conclusion:**

In conclusion, post-traumatic HLA-DR expression on monocytes is significantly reduced after multiple trauma and it is back to normal on day 14. No significant changes in HLA-DR expression on monocytes from severely injured patients suffering from SIRS, MODS or sepsis compared to those who did not have complications could be detected. Nevertheless, HLA-DR expression on monocytes may be used to identify the immunological pro- or anti-inflammatory phase the patient is going through.

## Background

Sepsis, Systemic Inflammatory Response Syndrome (SIRS) and Multiple Organ Dysfunction Syndrome (MODS) remain the most frequent causes of complications and death in severely injured patients. Authors could detect a malfunction of the immune system after major trauma as one reason for that [1]. The efficient function of the immune system is essential for an organism to fight pathogens. The innate immune system represents the first line of host defence. It performs phagocytosis as well as releases cytokines, thus activating the adaptive immune system. Surgical stress and trauma also lead to an activation of the innate immune system [2, 3]. In this case, so-called danger-associated molecular patterns have been identified as danger signals that mediate the early post-traumatic inflammatory response [2, 3]. Both pathogen- and danger-associated molecular patterns are recognized by immunologically competent cells [4]. HLA-DR molecules play a central role in the specific immune response, as they are required for antigen presentation and activation of helper T lymphocytes. These molecules are expressed on the surface of professional antigen-presenting cells such as macrophages or dendritic cells. Several studies have shown that HLA-DR expression on monocytes decreases after major injury, surgery and organ transplantation [5, 6]. Reduced HLA-DR expression on monocytes was considered to correlate with infectious complications and the development of sepsis.

One critical aspect following trauma is the ability of the organism to present MHC II antigens (HLA-DR) on monocytes, because the T helper cells will only react to foreign antigens that are presented on the macrophage surface using HLA-DR. Studies could show that in patients with an uneventful recovery from severe trauma or surgery, the level of monocyte HLA-DR expression fell within hours of trauma or surgery, but returned to normal within 1 week [6, 7]. In contrast, 3 weeks were required for HLA-DR expression to return to base level in those who developed infection, but finally recovered. Interestingly, in those who developed infection and sepsis and died as a result, HLA-DR expression decreased and never regained base levels.

Numerous markers of immune failure have been described in patients. Among these, the decreased expression on circulating monocytes of HLA-DR measured by flow cytometry has repeatedly been described as a robust marker of immune dysfunctions in septic shock patients [5]. So far, not many data exist about the association between HLA-DR expression on monocytes of severely injured patients and the development of a post traumatic SIRS, or MODS. Several studies are published regarding the HLA-DR expression and development of sepsis in patients after abdominal surgery or during long ICU stay; only one publication exists concerning the HLA-DR expression in polytraumatized patients with sepsis [8].

Up to now, the post traumatic kinetics of HLA-DR expression levels on monocytes after polytrauma has sparsely been shown. Moreover, it is not completely clear whether the HLA-DR expression on monocytes of polytraumatized patients is different on those who develop sepsis, SIRS or MODS during the post-traumatic course. As the early detection of the patients at risk for development one of these highly feared complications is most important for early treatment, this could be of great importance for the treatment of those patients during ICU stay. Therefore, the aim of this study was to analyse the HLA-DR expression on monocytes from polytraumatized patients during a 14-day post-traumatic course. Furthermore, it was the aim to investigate whether these kinetic changes were associated with the development of sepsis, SIRS or MODS.

## Patients and methods

### Patients

Consecutively, 24 blunt trauma patients were included in the study. Inclusion criteria were primary admittance to our department and Injury Severity Score (ISS) >16. Criteria for the exclusion of patients were patients younger than 18 or older than 65 years, admission >8 h after injury, penetrating injuries, pregnancy, premedication with immunosuppressive agents, and underlying cardiac, pulmonary, hematologic, or immunologic diseases. A control group of surgery patients undergoing total hip replacement was analysed as well. 2.7 ml EDTA blood samples were taken in both groups on admission, on days 3, 7 and 14. Blood sampling was conducted in accordance with the Declaration of Helsinki in its latest amendment. The local ethical institutional review board approved the study protocol (Nr. 2167). Informed and written consent was obtained from all trauma patients, all healthy volunteers and all control group patients or from their relatives.

Moreover, blood was taken from healthy individuals to determine a physiologic value of HLA-DR expression without trauma.

### Sepsis, SIRS, MODS

Existence of sepsis, SIRS or MODS was checked every day within the first 2 weeks after trauma. For the diagnosis “sepsis”, SIRS should be present and candida or bacteria had to be found in the systemic circulation.

For the existence of a SIRS, two of the following criteria had to be fulfilled [9, 10]:

1. Body temperature >38 or <36 °C
2. Heart rate >90 per minute
3. Breathing frequency >20 per minute or PaCO_2_ >4.3 kPa
4. White blood count >12,000 per μl or <4000 per μl or more than 10 % immature cells

For detection of MODS, the GORIS score was chosen. In this scoring system different points are given for the function of the seven organ systems (lung, heart, kidney, liver, blood, gastrointestinal tract, central nervous system). A normal organ function is scored with zero points while a total organ dysfunction gets 2 points [11]. Patients with a GORIS score of seven or more points on three consecutive days were considered to suffer from MODS.

### HLA-DR on monocytes

50 µl of the blood sample was mixed with 20 µl of antibody cocktail, which consisted of anti-HLA-DR PE and anti-CD14-CD64-PerCP-Cy5.5. (Becton–Dickinson, San Jose, CA, USA) in a FACS tube (Becton–Dickinson, Franklin Lakes, NJ, USA). This mixture was incubated at room temperature for 30 min. Then 45 µl of FACS Lysing Solution (Becton–Dickinson, San Jose, CA, USA) together with 405 µl distilled water was added and incubated for at least 10 min. After that time, the erythrocytes were lysed and a suspension of leukocytes was present. This suspension was used for flow cytometric analysis. After gating of the monocytes in the PerCP-Cy5.5 canal, a frequency bar chart of the PE canal’s intensity was generated. On the basis of the frequency bar chart, the mean intensity of each sample was analysed. Afterwards, on the basis of the mean intensity of each sample, the mean number of HLA-DR molecules on each monocyte could be determined by using a calibration series, which revealed the number of PE marked molecules per analysed mean intensity on the monocytes.

Quantification of the monocytic HLA-DR was performed using the program Cell Quest Pro.

### Statistical analysis

Results are presented as mean ± SEM. A one-way analysis of variance (ANOVA) and a multivariant Wilks’ Lambda were performed to determine significant differences between experimental means. A *p* value of <0.05 was considered statistically significant.

## Results

### Patients

24 polytraumatized patients were enrolled, of which 14 were males and 10 females. The mean age was 34 ± 2.7 years, ranging from 18 to 62 years. All patients survived the observation period of 14 days. The ISS was 28.4 ± 7.5.

A total of eight patients suffered from sepsis (33.3 %) (six males, two females), mean age was 37.3 ± 5.6 years, mean ISS was 27.8 ± 10.4 points and 18 patients suffered from SIRS (70.9 %) (11 males, 7 females), mean age was 32.4 ± 2.9 years and the mean ISS was 28.5 ± 8.2 points. MODS was present in five patients (20.8 %), three male and two female patients, the mean age was 33.6 ± 4.6 years and the mean ISS was 24.4 ± 5.9 points. In four of these five patients the MODS developed subsequent to sepsis. More detailed patient information is summarized in Table 1.

**Table 1:** Demographic data of the severely injured patient collective

Gender	Age (years)	Diagnosis	MODS	SIRS	Sepsis
Male	37	Craniocerebral injury II°, Rip fractures, lung contusion, upper spine fractures, thigh fracture	No	Yes	No
Male	19	Craniocerebral injury I°, fracture of the mandible, spleen rupture, tibialhead fracture, lumbal vertebrae fracture	No	Yes	No
Male	18	Craniocerebral injury I°, lung contusion, hip fracture	No	No	No
Female	28	Craniocerebral injury II°, rip fractures, lung contusions bilateral, spleen rupture, kidney contusion, vertebrae fractures	No	Yes	No
Male	49	Craniocerebral injury II° with brain contusion, rip fractures, haemato and pneumothorax, lung contusion, spleen rupture	Yes	Yes	Yes
Female	49	Craniocerebral injury III°, intracerebral bleeding, orbita and maxilla fractures, lung contusions bilateral, monteggia luxation fracture with open upper arm fracture	No	No	No
Female	22	Craniocerebral injury II°, rip fractures, lung contusions bilateral, lower leg fractures bilateral, forearm fractures bilateral	No	Yes	No
Female	41	Craniocerebral injury II°, serial rip fractures, lung contusions bilateral, bilateral tibia fractures, bilateral forearm fractures	No	Yes	No
Male	31	Craniocerebral injury with skull fracture, open patella fracture rip fractures, haemato and pneumothorax, lung contusions bilateral, acetabulum fracture,	No	No	No
Female	30	Craniocerebral injury II°, lung contusions bilateral, thoracic spine fracture, acetabulum fracture	Yes	Yes	No
Female	53	Craniocerebral injury I°, lung contusion, rip fractures, pneumothorax, liver contusion, open tibial head fracture, open patella fracture, anterior crucial ligament and posterior crucial ligament rupture, pelvis fracture	No	Yes	Yes
Male	27	Fracture of the mandible, nose fracture, kidney rupture, pelvis fracture, acetabulum fracture, forearm fracture right, open lower leg fracture right, fibula fracture left	No	Yes	No
Male	62	Craniocerebral injury I°, cheekbone fracture, rip fractures, haematopneumothorax, spleen and liver rupture, acetabulum fracture, thoracic spine fracture, pelvic fracture, retroperitoneal haematoma	No	Yes	Yes
Male	18	Craniofacial fractures, lung contusion, fibula fracture, posterior crucial ligament rupture, ankle fracture, calcaneus fracture radius fracture, ulna fracture	No	Yes	Yes
Male	24	Rip fracture right, thoracic vertebral fractures, open upper thigh fracture left, open lower leg fracture with rupture of the arteria tibialis posterior left, calcaneus fracture left, open upper arm fracture right, open ankle fracture right	No	Yes	No
Male	31	Pneumothorax right, spleen rupture left, kidney rupture left, pelvis fracture bilateral with rupture of the urethra, lumbal vertebral fracture, foot fracture right	No	No	No
Male	59	Le Fort fracture III° left, Le Fort fracture II° right, lumbal vertebral fractures, distal femur fracture right, open pilon-tibial fracture	No	No	No
Male	27	Craniocerebral injury I°, rip fractures 5-10 left, lung contusion left, spleen rupture, patella fracture	No	Yes	Yes
Male	36	Craniocerebral injury III° with open skull fracture, acetabular fracture, sacrum fracture, tibia fracture	Yes	Yes	Yes
Female	53	Craniocerebral injury II°, fracture of the sternum, serial rip fractures, haematopneumothorax bilateral, ileum fracture, thoracic spine fracture, fracture of the tibial head, contusio cordis	No	No	No
Male	32	Vertebral fractures in the thoracic and lumbal spine, olecranon fracture, serial rip fractures, lung contusions bilateral, spleen contusion, haematoma of the psoas	Yes	Yes	Yes
Female	30	Pneumothorax, spleen laceration, bilateral thigh fractures, patellar tendon rupture, pelvis fracture	No	Yes	No
Female	18	Craniocerebral injury II°, serial rip fractures, lung contusions bilateral, thigh fracture, liver contusion	No	No	No
Female	21	Haematopneumothorax, lung contusions bilateral, sacrum fracture, lumbal spine fracture, blunt abdominal trauma	Yes	Yes	Yes

### Control patients

The mean age of the 8 surgery control patients was 69 ± 4.5 years, ranging from 42 to 84 years. Five patients were males and 3 were females.

### Healthy volunteers

Besides, 9 healthy volunteers between 23 and 35 years (26.2 ± 1.2 years) were enrolled. Of these, 6 were males and 3 were females. A blood sample was taken once and the MFI was taken as blank value for all measuring points of the polytrauma patients and the controls, respectively.

### Mean fluorescence intensity (MFI) in polytrauma, control and healthy volunteers

**Fig. 1:**
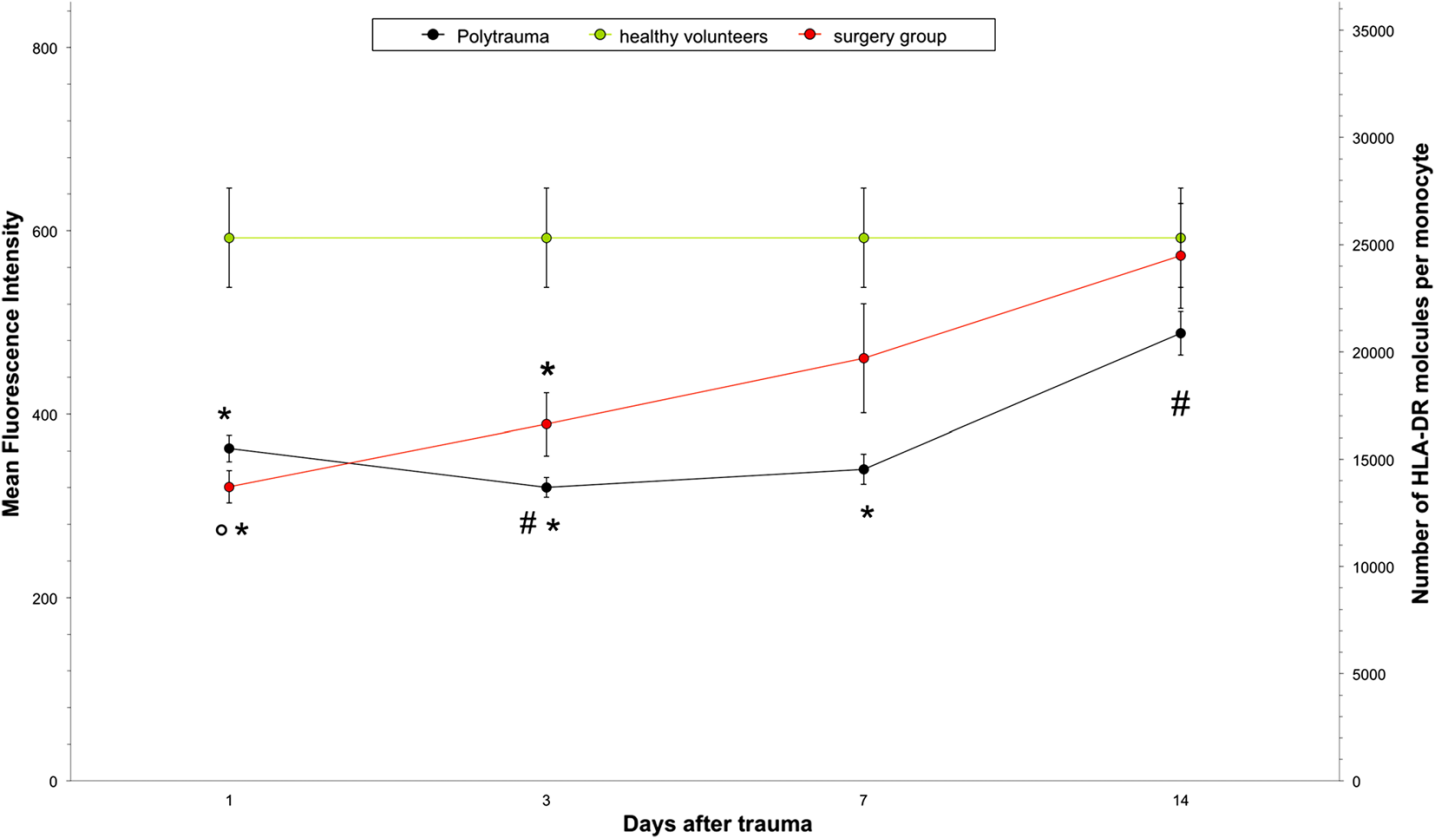
HLA-DR expression on monocytes from polytraumatized patients (*n* = 24), patients after total hip replacement surgery (*n* = 8) and healthy volunteers (*n* = 9). *p < 0.001 polytrauma patients and surgery group vs. healthy volunteers post trauma and days 3 and 7. °*p* < 0.05 surgery group post trauma vs. days 3, 7 and 14. Polytrauma patients: ^#^ *p* < 0.01 significant decrease day 3 vs. post trauma. ^#^ *p* < 0.0001 significant increase day 14 vs. post trauma (one way ANOVA, Wilks´ Lambda)

On day 1 after trauma, the MFI of all trauma patients was 362.8 ± 14.6. Compared to day 1, the MFI significantly decreased on day 3 (320.3 ± 10.7, *p* < 0.01) and increased again but not significantly on day 7 (340 ± 16.3). In the following course, MFI significantly increased further on day 14 (488 ± 23.8; *p* < 0.0001). On day 1, the MFI value of the surgery control patients was 319.7 ± 17.9. The control group showed a steady and significant increase of the MFI during the observation period, with values of 388 ± 34.7 on day 3 (p = 0.03), 460.4 ± 60 on day 7 and 572.8 ± 57.7 on day 14 (*p* < 0.05). The mean MFI of the healthy group was 592.3 ± 54.3. The surgery and polytrauma patients showed a significant decrease of the HLA-DR expression on day 1 compared to the healthy control group. While the HLA –DR expression in the surgery control group could already on day 7 reach a level close to baseline, the trauma patients needed 14 days (Fig. 1).

### MFI in MODS

Five patients developed a MODS during the observation period, whereas 19 patients had normal organ functions.

**Fig. 2:**
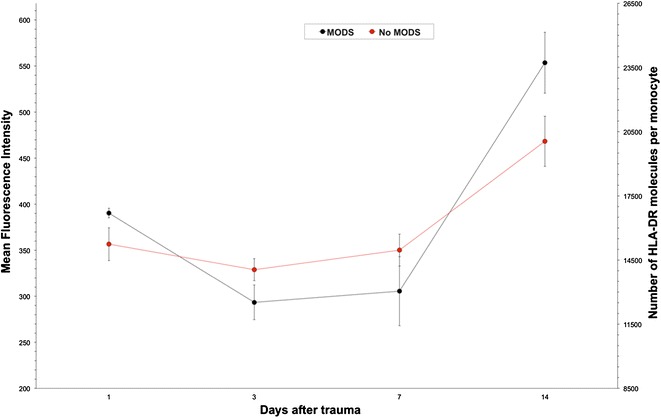
Patients with MODS (*n* = 5) showed decreased HLA-DR expression levels on monocytes compared to those who did not develop MODS (*n* = 19). Approximately on day 10 post trauma HLA-DR expression levels are alike

Patients with MODS showed the same trend as the overall polytrauma collective. MFI in patients with MODS was trend wise more decreased on day 3 and 7 compared to patients without MODS. However, differences were not significant (Fig. 2).

### MFI in SIRS

**Fig. 3:**
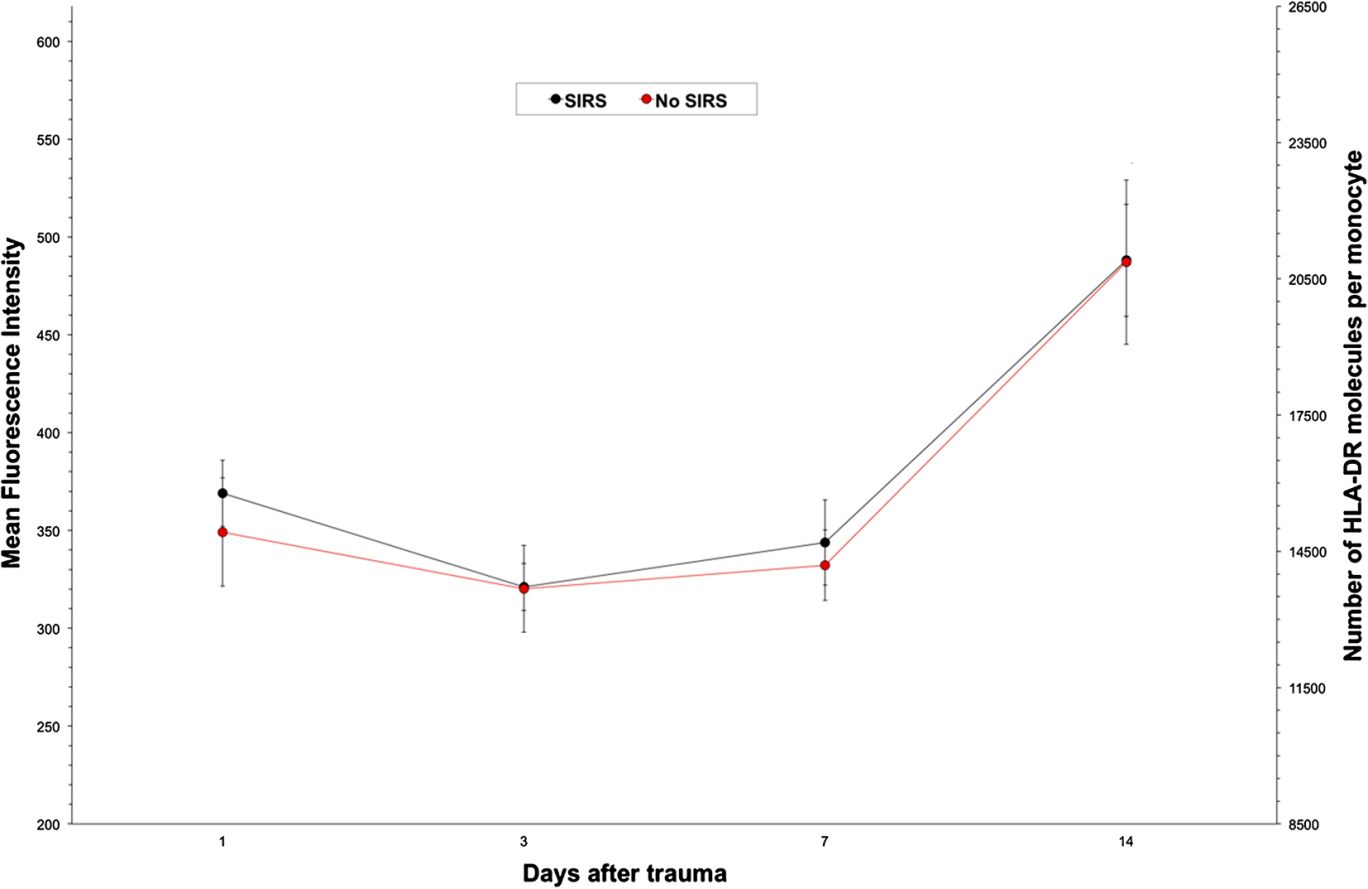
HLA-DR expression on monocytes from polytraumatized patients suffering from SIRS (*n* = 17) and those who did not develop a SIRS (*n* = 7). As nearly 70 % of the analysed collective developed SIRS, the *curves* are almost identical

From the twenty-four polytraumatized patients, seventeen were suffering from SIRS. The MFI was reduced on day 3 and increased significantly on day 7 and 14, respectively. Patients without SIRS showed the same post-traumatic course of MFI with similar values (Fig. 3).

### MFI in sepsis

**Fig. 4:**
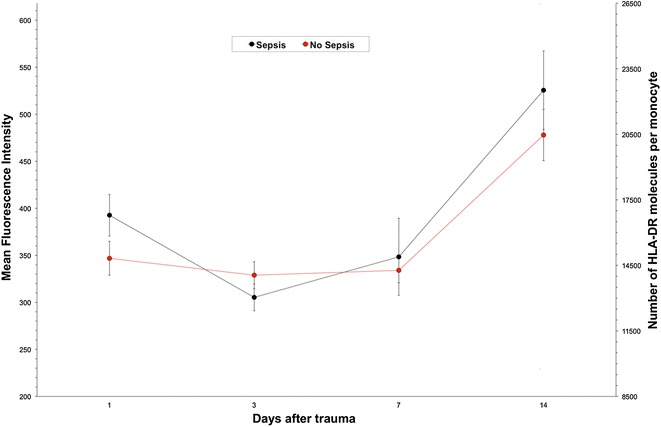
Patients with sepsis after polytrauma (*n* = 8) showed a sudden decrease of monocytic HLA-DR expression levels immediately after trauma while patients without sepsis (*n* = 16) could hold a steady expression level. After 7 days both groups are back on one similar level

Eight out of the twenty-four polytraumatized patients developed sepsis during the post-traumatic course. In the systemic circulation of all septic patients, bacteria (haemolytic staphylococcus, staphylococcus epidermidis, enterobacter aerogenes) or candida could be detected. Patients suffering from sepsis showed decreased MFI values on day 3 and 7 post-trauma with a significant increase on day 14 compared to patients without sepsis. In contrast to the patients with sepsis, the MFI reduction on day 3 and 7 was only marginal in patients without sepsis while the significant increase on day 14 could be detected as well (Fig. 4).

## Discussion

In the present study, the post -traumatic kinetic of HLA-DR expression on monocytes after polytrauma compared to surgery and healthy volunteers has been analysed. Moreover, the use of HLA-DR expression on monocytes as a prognostic marker for development of MODS, SIRS and sepsis in polytraumatized patients has extensively been researched and analysed. A significant decrease in HLA-DR expression on monocytes during the first 14 days after severe trauma could be detected. This result comes along with the findings of several other authors analysing HLA-DR levels after surgery or organ transplantation [12–15]. Persisting low HLA-DR values were found in patients who subsequently developed secondary infections, whereas HLA-DR levels increased generally within 1 week in individuals who recovered uneventfully [6 , 8, 16, 17]. Previously, HLA-DR expression was usually assessed as a predictor of septic complications after trauma, surgery or pancreatitis [6, 8, 16, 17], but no statements concerning MODS or SIRS were made so far. Only one study deals with sepsis development in polytraumatized patients and HLA-DR expression on monocytes but in this study neither MODS nor SIRS development was analysed. Moreover, neither the healthy HLA-DR expression in volunteers nor the HLA-DR expression after surgery had been analysed as control groups. In contrast, Ditschkowski et al. separated the polytrauma collective in three groups depending on the severity of injury [8]. In contrast to Ditschkowski et al. no significant differences between the HLA-DR expression of polytrauma patients with sepsis and without sepsis could be found in this study. One reason for this might be the patient number. While Ditschkowski et al. enrolled 77 patients of whom 20 developed a sepsis, we could include 24 patients of whom eight developed a sepsis. Although we have enrolled less patients, the sepsis rate of both collectives was more or less 30% . The HLA-DR expression curves in our study looked similar to the ones from Ditschkowski et al. and they followed the same trend [8].

Other authors could also show a correlation between a decreased HLA-DR expression on monocytes and the development of a sepsis during the post-operative course [18–20]. These data are not comparable with the presented results, as they have analysed different patient groups like burn patients or patients suffering from abdominal or vascular surgery. Nevertheless, a trend towards lower HLA-DR expression could be observed in our sepsis patients.

We believe that polytrauma patients cannot be compared with patients suffering from sepsis due to nosocomial infections, burns or abdominal surgery complications because the immunological processes after a polytrauma are different. Due to polytrauma, the patient has to face the first immunological hit (e.g. hypoxia, hypotension, organ and soft tissue injuries, fractures), followed by a second hit (e.g. ischaemia/reperfusion injuries, operative interventions, infections). This induces a host defence response, which is characterized by local and systemic release of pro-inflammatory cytokines, complement factors and acute phase proteins, as well as hormonal mediators. In parallel, anti-inflammatory mediators are produced (compensatory anti-inflammatory response syndrome, CARS). An imbalance here mayo be responsible for organ dysfunction and increased susceptibility to infections.

Therefore, the HLA-DR expression may be dependent on the pro- or anti-inflammatory phase the individual is passing through at the moment of blood collection. SIRS is defined as a hyper-inflammatory phase and, therefore, associated with normal or raised HLA-DR expression [21].

Later during the anti-inflammatory phase, CARS, the production of anti-inflammatory mediators is increased and monocytes downregulate the HLA-DR expression [21]. Especially, IL-10 causes an internalization of HLA-DR thus decreasing the HLA-DR expression on monocytes. This can be prevented by anti-IL-10 treatment. Thus, in individual patients, profound CARS is associated with low numbers of HLA-DR expressing monocytes. However, in other patients the blood sample might have been taken while the individual was in the SIRS phase, in which case monocyte HLA-DR may be normal or raised whatever the prognosis of the patient. Measurements of monocyte HLA-DR expression may be worth pursuing as one possible indicator as to whether the patient is in an inflammatory or anti-inflammatory phase.

Some studies exist analysing the different impact of ageing on monocytic HLA-DR expression. Seidler et al., for example, could show a decrease of monocyte HLA-DR expression in aged healthy volunteers (>50 years). The difference was significant compared to the very young control group (<30 years) but it was not significant compared tot he middle aged (>30 years) [22]. Others did not find any correlation with HLA-DR expression and age [23]. Therefore, we do not think that the age difference between our study group (ca. 30 years) and the surgery group (ca. 69 years) is crucial for our results.

20 % of our severely injured patients developed MODS during the post traumatic course, which is comparable with results from other studies, analysing patients with blunt abdominal trauma, with MODS rates between 7.9 and 26.1 % [24].

Decreased expression of HLA-DR on monocytes is a hallmark of altered immune status in patients with SIRS. West et al. could show that HLA-DR expression on monocytes correlates with injury severity [25]. They could also show decreased monocytic HLA-DR levels 48 h after trauma as others have shown before [25, 26]. This comes along with our results. Nevertheless, we could not detect significant differences of HLA-DR expression on monocytes of patients suffering from SIRS and those who did not after multiple trauma.

Kim et al. could detect that HLA-DR expression is differently modulation on CD14^HIGH^ (classical) and CD14^LOW^ (inflammatory) monocytes after systemic inflammation.This shows that also the HLA-DR expression on monocyte subpopulations is involved in the development of a SIRS [27].

There are several limitations to this study. First, the group size of 24 polytraumatized patients is very small. This is due to the fact that the analysed patient population, traumatized patients with an ISS >16 points, is a rare entity. Nevertheless, more studies with a bigger collective are needed. Moreover, HLA-DR expression on monocytes was analysed only at some time points (directly after trauma, 3, 7 and 14 days post trauma). Therefore, significant changes in between might be missed. However, the time curve after severe trauma and the important time points have been extensively studied during the past decades and most immune responses are detected during the first 14 days with a peak directly after trauma up to 3 days postop [28].

In conclusion, post-traumatic HLA-DR expression on monocytes is significantly reduced after multiple trauma. HLA-DR levels are at 14 days normal. No significant changes in HLA-DR expression on monocytes from severely injured patients suffering from SIRS, MODS or sepsis compared to on those who did not have complications could be detected. Nevertheless, HLA-DR expression on monocytes may be used to identify the immunological pro- or anti-inflammatory phase the patient is going through in combination with other immunological markers. More studies with a larger polytrauma collective are needed to confirm and widen our knowledge about post-traumatic immune system dysfunctions.

## Abbreviations

HLA-DR: human leucocyte antigen-DP, DQ, DR
SIRS: systemic inflammatory response syndrome
MODS: multiple organ dysfunction syndrome
MOF: multiple organ failure
FACS: fluorescence-activated cell sorting
CARS: compensatory anti-inflammatory phase
ISS: injury severity score
ICU: intensive care nit
MFI: mean fluoresceunce intensity
TLR: toll-like receptor
